# Exploring the factors influencing academic learning performance using online learning systems

**DOI:** 10.1016/j.heliyon.2024.e32584

**Published:** 2024-06-06

**Authors:** Ashraf Bany Mohammed, Mahmoud Maqableh, Dhia Qasim, Faisal AlJawazneh

**Affiliations:** a School of Business, University of Jordan, Amman, Jordan; b Faculty of Business, Alzaytoonah University of Jordan, Amman, Jordan

**Keywords:** Online learning, Academic learning performance, Quality, Student satisfaction, Task-technology fit

## Abstract

-Online learning has become vital and fundamental for learning success in higher education institutions to cope with various learning methodologies or crises such as COVID-19. Therefore, this study investigates factors influencing the academic learning performance among diploma and bachelor's degree students who utilize online e-learning systems in Jordan. Using a conceptual model developed from an extensive previous literature review, the study used a quantitative research methodology to collect data via survey questionnaires. Using PLS-SEM, the empirical analysis of 846 questionnaires collected from Jordanian students demonstrates that task-technology fit and students' satisfaction positively influence learning performance and contribute significantly to student academic success using online learning systems. While the quality of system, service, and information are critical determinants of task-technology fit, student interaction, and instructor interaction are critical determinants of student satisfaction. The study highlights the importance of user-friendly interfaces, effective communication channels, collaboration, and positive learning environments. The findings provide practical insights for universities, online learning system designers, administrators, and instructors to enhance student-learning outcomes. Additionally, system providers can use these findings to address essential successful tools and features needed to create effective and efficient online learning environments. This study highlights practical implications that support higher education institutions' efforts to enhance their online e-learning systems quality and improve student-learning outcomes while helping system providers address essential successful tools and features needed.

## 1 Introduction

Students and scholars have interchangeably used online learning as e-learning, distance, and blended learning [1]. It is known that launching and using of online learning started in 1995, as it was, the first dedicated to uploading necessary study documents; afterward, it evolved to be a blackboard [2]. During the past decade, especially during the COVID-19 pandemic, plenty of lockdowns were made to reduce the virus's negative impacts, raising the need for students to attend lectures from home [3,4]. Marinoni et al. (2020) have mentioned that according to UNESCO, 185 countries were forced to fully adopt online learning, which had a global adverse influence on education and interaction quality between students and their scholars [5]. The sudden conversion to a completely online learning environment was a challenge that should be faced by all parties involved in academia due to the digital divide between students and their teachers [6,7].

Research has emphasized both the benefits and drawbacks of online education; the fact that it provides flexibility in which any student with an internet connection and a browser can enter the online lecture is considered a significant advantage in online learning. Additionally, they have highlighted the extent to which online education is crucial in cutting costs for students who cannot afford to visit a school or university daily [8]. Furthermore, it is inevitable to have countries planning for different scenarios in accordance with the condition and spread of the pandemic, so it was really of high importance to implement solid and outstanding governmental policies to be strictly followed by all academic institutions [9]. Additionally, based on a qualitative study covering 19 countries in the MENA region, it has been concluded that for the interest of both students and faculty members to implement and use blended learning within academic institutions to be able to coop between face-to-face interaction and technology usage as well [10].

### 1.1 Online learning in Jordan

Although known for its resource limitations, Jordan is known for its outstanding higher education quality and standards with a total of 40 universities, including public, private, university colleges, and one regional university, which underlines the crucially of implementing online learning to accommodate massive numbers of students’ post-COVID-19 [11,12]. It was clear that Jordan is considered a leading country in implementing technology, especially in their academic institutions; this was done through long-term partnerships with different entities and bodies [13]. Many research works investigated different facets of internet-based education pre and post the outbreak of the pandemic [14]. Al-shboul and Alsmadi (2010) highlighted the potential of Moodle platforms in Jordanian universities [15]. In recent years, Jordan has approached online learning cautiously, gradually implementing the necessary technology and infrastructure to allow students and scholars to take full advantage of it [16].

However, research on online learning within Jordan Post-COVID-19 suggests that undergraduate students have faced numerous obstacles from different aspects [17]. Alsoud and Harasis (2021) found that students were often anxious about losing their internet connection, facing financial obstacles when purchasing a device to access lectures [18]. Nonetheless, it is recommended that schools and universities should take full responsibility to promote student participation in online education amid the COVID-19 pandemic, as facilitating conditions might impact students’ viewpoints regarding online learning [14,19]. The significance of extending research on online learning from different models, factors, and research designs within the Jordanian context cannot be overstated, especially given that it is a developing country.

### 1.2 Theoretical gap

Recent research works have demonstrated universal acceptance, broad applicability, and TTF (TTF) of the Information Systems (IS) Success Model in the information systems domain [8]. Awad et al. (2022) built on the IS success model by adding “relative advantage, self-efficacy, and COVID-19 perceived risk” to measure the continuous usage intentions of E-learning in Jordan [20]. Al-Nassar (2020) used system and information quality derived from the IS success model in an attempt to measure mobile learning success in the Jordanian context [21]. Alyoussef (2023) combined the IS success model with perceived ease of use, usefulness, TTF, and enjoyment to measure e-learning benefits within academic institutions [22]. Wu and Chen (2017) integrated the Technology Acceptance Model (TAM) with TTF to measure the continuous usage intentions of MOOCS [23]. Aldholay et al. (2018b) also combined the IS Success model with TTF to measure performance impact in the context of Yemen, dividing it into efficiency and effectiveness [24].

Our proposed model integrates the IS success model and its impact on TTF with important educational factors such as interaction with colleagues and instructors to measure student satisfaction and learning performance in the Jordanian context. Furthermore, up to the authors' knowledge, our study is the only one investigating the influence of mediation of student satisfaction on the relationship between TTF and learning performance. Our research investigates factors influencing the academic performance of diploma and bachelor's degree students using online e-learning systems in Jordan. Specifically, we aim to combine the main elements of the Delone & Mclean IS success model with elements that might increase or inhibit academic performance, such as interaction with colleagues and instructors. Additionally, our study seeks to uncover insights regarding the impact of the IS success model elements on TTF, as well as the influence of interaction with colleagues and instructors on student satisfaction, leading to the influence of TTF on student satisfaction and ultimately the influence of both on academic performance.

In the remainder of this study, we will comprehensively analyze the factors affecting online learning performance, first, providing an extensive review of the relevant body of literature and establishing the conceptual framework for our study. Then, we describe our research methodology and data collection process. Afterward, the empirical findings and statistical analysis are presented, focusing on the influence of various factors on online learning performance. In the next section, we will present and examine the findings from our study analyses and offer recommendations for educators and institutions. Finally, we conclude our study by summarizing critical practical and theoretical implications and suggest future research directions. Throughout the article, we aim to provide a comprehensive investigation of factors that influence online learning performance, offering fruitful insights for both academia and educational practitioners.

## 2 Review of existing literature and development of hypotheses

The development of our conceptual model explores how various factors influence TTF and subsequently affect students' satisfaction and online learning performance. This model is founded based on the IS Success Model [8], which suggests that supporting users' tasks is a crucial element of information systems success. By combining IS Success Model and TTF, we expect our conceptual model to guide users in utilizing technology to achieve their tasks, such as how it could help students enhance their online learning performance. TTF is based on three main domains: Technology Characteristics, Task Characteristics, and Individual Characteristics [23,25]. These domains include various attributes such as interactions with colleagues and instructors, quality of systems, information, and services which collectively contribute to the fit between technology and tasks [26].

Technology Characteristics including quality of systems, information, and services, capture both the technical and functional features of the system. These dimensions influence users' perceptions of the system's reliability, relevance, and usability, affecting their ability to perform tasks effectively [27]. Interaction with colleagues and instructors, on the other hand, represents social aspects of technology use, which are included within Individual Characteristics. These interactions promote collaboration, support, and engagement, ultimately enhancing users' satisfaction and performance [28,29]. Therefore, TTF plays a crucial role in our model by mediating the relationships between quality of systems, information, and services, on one hand, and students' satisfaction and online learning performance, on the other. Our model highlights the significance of TTF as a critical determinant of success in online learning environments, providing a comprehensive framework for understanding and evaluating the efficacy of educational technologies.

### 2.1 System quality

In information processing technology, system quality is crucial [27]. According to Delone and Mclean (2003), it is closely linked with technical success [30]. Moreover, the system's interface should be user-friendly to grant end-users a chance to quickly learn and interact with it, as Yuce et al. (2019) emphasize [31,32]. This implies that system quality is a benchmark for shaping end-users’ perceptions and evaluating the system accordingly. In addition, system quality helps integrate functional aspects with the tasks assigned to end-users [26].

Furthermore, Wang and Liao (2008) highlight the importance of regularly maintaining, checking, and assessing the system for errors and bugs [33]. This is a crucial step towards achieving users’ desired goals. According to Yuce et al. (2019) and Chen et al. (2022), system quality positively impacts TTF [32,34]. Therefore, we hypothesize that system quality is vital in ensuring the system is efficient and effective, ultimately leading to a positive user experience.

H1 *System Quality positively influences on Task-Technology Fit*.

### 2.2 Information quality

Information quality is a critical aspect of any information system, regarding the relevance, timeliness, and accuracy of the generated information [35]. The success of a system's output is closely tied to the quality of the words it generates to enhance the user's learning experience [31,32]. It is essential that an information system can generate reliable, robust, effective, and relevant knowledge, as this is how the quality of the output is measured [27]. Wu and Chen's (2017) research highlights that high information quality can significantly improve a user's ability to complete assigned tasks [23]. Further studies by Yuce et al. (2019), Candrawati et al. (2023), and AlJukhadar et al. (2014) have concluded that information quality has a positive impact on TTF [32,36,37]. Therefore, we could hypothesize that.

H2 *Information Quality positively influence on Task-Technology Fit*.

### 2.3 Service quality

The service quality in information systems is defined as the level of support that users receive, according to Ref. [38]. Rana et al. (2015) further elaborated that service quality is centered around the support that end-users receive from the IT personnel [35]. To measure service quality, various elements including responsiveness, precision, dependability, technical skill, and empathy are taken into account, derived from the SERVQUAL theory in the marketing domain [39].

It is noteworthy that service quality highly impacts students' learning experience and can be a benchmarking approach for expected and provided service [32]. Heinze and Matt (2018) emphasized the importance of service-technology fit, where the quality of service delivered aligns with the resources available, such as time, speed, and location [40]. The study also highlighted the significance of aligning assigned tasks with the quality of technologies provided to students, which can enhance their mental capabilities. Yuce et al. (2019) and Chen et al. (2022) have concluded that service quality positively impacts TTF [32,34]. Therefore, we could hypothesize that.

H3 *Service Quality positively influence on Task-Technology Fit*.

### 2.4 Interaction with colleagues

Students' positive perception of their learning experiences constitutes student satisfaction [41]. Lu and Chiou (2010) contend that various theoretical models can be integrated to measure student satisfaction using e-learning systems, emphasizing the usefulness of both TAM and IS success models [42]. Alyoussef's (2021) study highlights the significance of task technology fit in influencing student satisfaction and adoption of learning technology [43]. Therefore, e-learning systems must align with the tasks students are required to do and facilitate the learning process to ensure a positive experience that encourages continued use of the system. Lin (2012) found that perceived technology fit plays a role in student satisfaction, with TTF being a crucial factor in overall satisfaction [44].

Oyarzun et al. (2018) concluded that implementing learning technologies that facilitate collaboration and cooperation among colleagues in a study setting is essential to satisfaction [28]. This is because exchanging thoughts and experiences through online learning interaction builds a healthy learning environment that keeps students motivated and productive, initially driven by their satisfaction [12]. In the context of high schools, numerous researchers have reached different conclusions regarding how interaction among colleagues leads to student satisfaction. For instance, Borup et al. (2013) and Yu et al. (2020) have mentioned that learner-learner interaction builds course satisfaction [45,46]. However, Zhang and Lin (2020) contradicted the results of Borup et al. (2013) by concluding that such an interaction does not influence course or student interaction [45,47].

H4 *Interaction with Colleagues positively influence on Students' Satisfaction*.

### 2.5 Interaction with instructors

According to Moore's research (1989), instructors and students exchange communications, thoughts, and experiences [48]. Instructors play a crucial role in this process by designing a course syllabus that aligns with course objectives, providing regular feedback on student performance, and offering continuous support and counseling to students regarding their progress [49]. Obviously, highly competent instructors using technology are best suited to deliver the best experience in this interaction [29,49].

Plenty of studies have found a direct positive relationship between student satisfaction and interaction with instructors. Kuo et al. (2013) discovered that interaction with instructors positively impacts student satisfaction, while Ali and Ahmad (2011) reached the same conclusion, finding that this interaction also affects course satisfaction [41,50]. These findings highlight the cruciality of establishing a positive and supportive learning environment through effective interaction between instructors and students [49]. Therefore, we could hypothesize that.

H5 *Interaction with Instructors positively influence on Students' Satisfaction*.

### 2.6 Task-technology fit (TTF)

TTF was developed by (Goodhue and Thompson, 1995), in which it states that the user will not adopt or use a particular technology unless it goes in line with tasks assigned to them and plays a role in enhancing their performance [51,52]. This concept asserts that users will not embrace a technology unless it matches their assigned tasks and improves performance. The extant literature shows that TTF has been implemented in different industries and studied extensively with different technologies. For example, Omotayo and Haliru (2020) have emphasized the impact of TTF on the use of digital libraries [53]. Meanwhile, Faqih and Jaradat (2021) have investigated the effects of TTF and the Unified Theory of Acceptance and Usage of Technology (UTAUT) on adopting augmented reality in education [54]. Therefore, the studies mentioned above illustrate the versatility of TTF in different industries and its alignment with various adoption and usage models, such as TAM, UTAUT, and the Technology-Organization-Environment (TOE).

Student satisfaction is students' positive perception of their learning experiences [55]. Lu and Chiou (2010) argue that various theoretical models can be incorporated to measure student satisfaction using E-learning systems, emphasizing the usefulness of both TAM and IS success models [42]. Alyoussef's (2021) study highlights the significance of task technology fit, which can significantly influence student satisfaction and adoption of learning technology [43]. E-learning systems must facilitate learning and align with students' tasks to ensure a positive experience and encourage continued use. Additionally, Lin (2012) found that perceived technology fit plays a role in student satisfaction, with TTF being a critical factor in determining overall satisfaction [44]. Therefore, we could hypothesize that.

H6 *Task-Technology Fit positively influence on Students' Satisfaction*.

Research conducted by Isaac et al. (2019) revolving around the online learning environment within the context of Yemen proposed and mentioned that TTF is crucial in achieving positive learning performance [8]. This is since TTF concerns the compatibility between the task assigned and the technology used to accomplish the task [25]. Additionally, it is good to know that research on this relationship, particularly, applies to different domains. Gatara and Cohen (2014) have used TTF as a mediator between mobile health tool usage and worker performance; the results proposed show a positive influence of TTF on worker performance within the Kenyan context [56]. Furthermore, Lee and Lehto (2013) have concluded that TTF positively impacts effectiveness, performance, and net advantages [57].

Various theories could be combined to measure student satisfaction in using and interacting with e-learning systems, as Lu and Chiou (2010) have stated [42]. They emphasized that both TAM and IS success models could be used to measure student satisfaction. In addition, Alyoussef (2021) has stressed the importance of task technology fit as a vital factor that strongly influences students’ satisfaction [43]. Students would not adopt any learning technology that does not make their learning process more accessible and support the assigned task [58]. This fit is crucial to ensure that students feel satisfied in interacting and using e-learning, which will result in the ongoing intention to use the system. Therefore, we propose the following hypothesis.

H7 *Task-Technology Fit positively influence on Online Learning Performance*.

### 2.7 Students satisfaction

In the theoretical framework section of academic articles, various theories can be combined to measure student satisfaction in using and interacting with E-learning systems. As Lu and Chiou (2010) have stated, both TAM and IS success models can be used to measure student satisfaction [42]. Alyoussef (2021) has emphasized the importance of task technology fit as a vital factor that strongly influences students’ satisfaction [58]. Students would not adopt any learning technology that does not make their learning process more manageable and support assigned tasks. Therefore, having this fit is crucial to ensure that students feel satisfied in interacting and using e-learning, which will lead to the continuous usage intention of the system.

Moreover, the perceived fit of technology is examined by Lin (2012), and user satisfaction is considered a crucial measure and indicator of the success of system adoption [31,44,59]. User satisfaction is also highlighted as a reflection of the user's enjoyment in a study conducted by Ref. [60]. It is found that user satisfaction positively impacts learning performance within an online learning context [58,61]. These findings support the conclusions drawn by Stefanovic et al. (2016), who have tested the same relationship within the E-government context [62]. However, the results proposed by the previously mentioned research by Isaac et al. (2017) and Stefanovic et al. (2016) do not go in line with the research done by Daud et al. (2011), which has reported that there is no significant impact of user satisfaction on performance impact [[61], [62], [63]]. Therefore, the following hypothesis is proposed.

H8 *Students' Satisfaction positively influence on Online Learning Performance*.

### 2.8 Online learning performance

Previous literature has thoroughly highlighted factors influencing student's learning performance with less emphasis on online learning environments, for instance: Sung et al. (2016) have focused on the integration of mobile devices in the learning environment to investigate their influence on student's performance [64]. However, Qureshi et al. (2023) have investigated the influence of student engagement on their learning performance [65]. This minimal focus on investigating student's performance on online platforms beyond COVID-19 stresses on the importance of our study.

The essence of our study revolves around online learning performance, as it is worth mentioning the research conducted investigating the online learning performance in the Jordanian context are limited, and this is due to the extensive focus on other measures such as Instructor quality, educational infrastructure, and learning self-efficacy [66]. Online learning performance focuses extensively on the behavioral aspect of students and the extent to which they managed to improve academically while being involved in online learning environments [66,67]. Finally, the evolution of online learning platforms, tools, and methodologies in Jordan have been noticeable especially beyond the COVID-19 outbreak which stresses on the extent to which the higher institutions in Jordan are considered vital and must coop with the most recent technological advancements to keep operating on the same pace [68].

Butt et al. (2022) have proposed that TTF influences online learning performance, and that stems from the fact that there should be an alignment between the online task required and the technology dedicated to have it achieved [69]. These results also emphasized by Liu et al. (2023) who have also targeted Jin University students and found that TTF Influences online learning performance, their assumption is based upon the importance of aligning task, technology, individual and environment along with each other to ensure having a positive learning performance outcome within online means of communication [70]. Furthermore, Butt et al. (2022) re-emphasized the results of their previous study done in 2021 and found the same influence to be true, but this time by focusing on the moderating role of cognitive absorption [71].

While our study focuses on online learning performance it is worth mentioning that satisfaction might act as an important predictor for online learning performance since satisfaction is highly related to student motive to learn, and the extent to which students and teachers are on good terms [72]. Furthermore, Farooq et al. (2011) have mentioned that student satisfaction should be the most factor when talking about enhancing their performance whether they are taking courses on campus, online, or on a blended basis [73]. Additionally, Wang (2022) stressed that student's online learning performance is a reflection of the extent to which the course is interactive and the module is taught in clear, concise, and understandable means [74]. Gopal et al. (2021) conducted a study among students studying Business Management and Hotel Management among Indian Universities during the pandemic, and have found that satisfaction influences online learning performance [75].

**Fig. 1:**
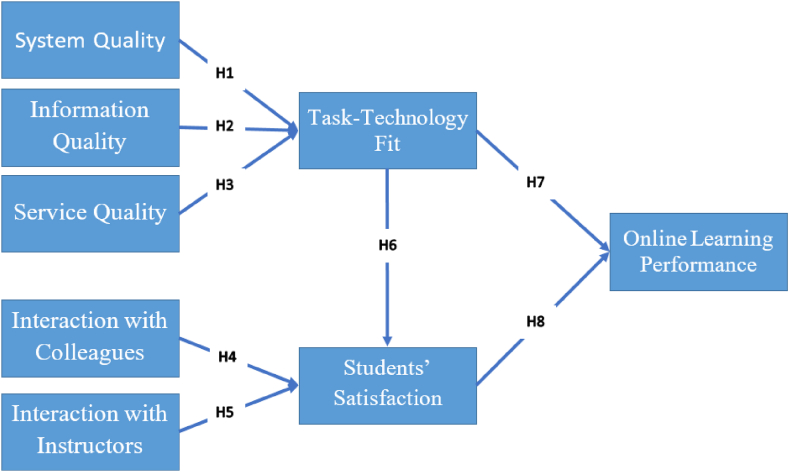
The proposed conceptual model.

Our proposed model presents several constructs that demonstrate how quality (system, information, and service) influences TTF [8]. Furthermore, it highlights the impact of interaction with colleagues and instructors on students' satisfaction [76]. Moreover, the model also examines how TTF and students' satisfaction impact the learning performance of students using online learning systems [31,61,76]. The proposed model also aims to investigate how student satisfaction mediates the relationship between TTF and student learning performance. The model presents eight hypotheses for testing, as shown in Fig. 1. Additionally, all the variables used in building the conceptual model are explained in Table 6 in Appendix A.

## 3 Research methodology

### 3.1 Instrument development

The proposed research model includes various constructs such as overall quality, interaction, TTF, satisfaction, and learning performance. These constructs have been operationalized based on measures from previous studies [31,61,76,77]. A 5-point Likert scale was employed to assess the questionnaire items, with responses ranging from "1 = strongly disagree" to "5 = strongly agree". The survey was accurately translated from English to Arabic as the respondents were Arabic speakers. Reverse translations were performed to ensure accuracy and consistency, following the guidelines of [78]. The initial instrument underwent evaluation through ten semi-structured interviews conducted with eLearning users and academics, resulting in several minor adjustments to the survey questions.

### 3.2 Data collection

Data were collected from the intended sample i.e., online learning users during the pandemic using an online survey. The data were gathered throughout the fall semester of the 2022/2023 academic year. Participants were randomly chosen among 5000 diploma and bachelor degree students from a university college in the Hashemite Kingdom of Jordan. The university college grants diplomas after two years of study and four-year bachelor degrees. The majority of the students are enrolled in diploma programs, with few studying bachelor programs. The online survey was published on the university college website, and students were free to fill out the survey, along with guidance on the purpose of the study and a prior informed consent of participation. Accordingly, this research obtained an approval from the ethical committee at the university college.

Determining the correct sample size for performing regression analysis is a topic that researchers are still uncertain about. O'Rourke and Hatcher (2013) suggest that a study should have a sample size of at least 100 participants or the number of constructs should be multiplied by five to obtain the required sample size [79]. As the questionnaire used in this study contained 29 items, the sample size required should be over 160 students. Hair et al. (2014 have suggested that an ideal sample size ranges from 100 to 200, while Krejcie and Morgan (1970) have required 351 participants from a population of 5000 [80]. Therefore, the collected surveys, which numbered 846, satisfy the necessary sample size criteria for hypotheses testing.

The respondents' characteristics of the study sample show that it is divided almost equally among males (49.6 %) and females (50.4 %), with ages ranging between (19–21) for around half of them (52.6 %). They are enrolled in their first year (50.5 %) and second year (42.8 %), and the majority of them (89.8 %) are coming from a diploma education background. As a result of that, approximately (53.3 %) have less than one year of experience in online learning systems, and (44.2 %) of them indicate (1–2) years of experience in similar online learning systems. The descriptive statistics of respondents’ characteristics for this study are summarized in (Table 1).

**Table 1:** Respondents’ characteristics.

Items	Categories	Frequency	Percentage
Gender	*Male*	420	49.6 %
	*Female*	426	50.4 %
Age	*17–19*	215	25.4 %
	*19–21*	445	52.6 %
	*22 years and above*	186	22.0 %
Education Background	*Diploma*	760	89.8 %
	*Bachelor's degree*	86	10.2 %
Study Level	*First-year*	427	50.5 %
	*Second-year*	362	42.8 %
	*Third-year*	51	6.0 %
	*Fourth-year*	6	0.7 %
Online Learning Experience (Year)	*Less than 1*	451	53.3 %
	*1–2*	374	44.2 %
	*More than 2*	21	2.5 %

## 4 Data analysis and results

The study's data analysis was carried out using SmartPLS (4.0) in a two-phase process. The first phase involves the measurement model and the second phase is the structural model. This involved evaluating path coefficients, statistical significance, model fit, and predictive relevance. Path coefficients indicate the strength and direction of relationships between the study constructs, while statistical significance is assessed using p-values. Additionally, model fit was assessed by examining various goodness-of-fit measures, such as the chi-square test, the normed fit index (NFI). Finally, predictive relevance was assessed by examining the R-squared values for endogenous constructs in the model.

### 4.1 Measurement model

This section of the report details the outcomes of the validity and reliability assessments conducted on the research instrument. All items' factor loading was higher than 0.7, as shown in Table 2, surpassing the acceptable level suggested by Ref. [81]. Cronbach's alpha (***α***) values for the study's constructs confirmed the internal reliability and consistency, ranging from 0.80 to 0.94, exceeding the accepted level of 0.70 for this research [82]. Moreover, all latent constructs' composite reliability (CR) values ranging from 0.91 to 0.96 were above the acceptable level of 0.70, establishing the reliability and consistency of the study's constructs [83]. Furthermore, the study confirms convergent and discriminant validity. The Average Variance Extracted (AVE) values for all constructs ranging from 0.78 to 0.90 were above the acceptable threshold level of 0.50 [83,84]. The appropriate convergent and discriminant validity level is indicated by higher cross-loading within the same construct than with other constructs, as shown in Table 3. These findings support the measurement model's reliability and consistency and demonstrate the validity of the study's constructs.

**Table 2:** Factor loadings for all items.

Items	INC	INI	IQ	LP	SAT	SEQ	SQ	TTF
INC1	*0.951*							
INC2	*0.955*							
INC3	*0.910*							
INI1		*0.942*						
INI2		*0.954*						
INI3		*0.917*						
IQ1			*0.902*					
IQ2			*0.912*					
IQ3			*0.906*					
IQ4			*0.912*					
IQ5			*0.920*					
LP1				*0.906*				
LP2				*0.925*				
LP3				*0.711*				
LP4				*0.930*				
LP5				*0.932*				
LP6				*0.917*				
SAT1					*0.954*			
SAT2					*0.949*			
SAT3					*0.955*			
SEQ1						*0.854*		
SEQ2						*0.905*		
SEQ3						*0.895*		
SQ1							*0.894*	
SQ2							*0.932*	
SQ3							*0.923*	
TTF1								*0.905*
TTF2								*0.958*
TTF3								*0.943*

**Table 3:** Constructs’ reliability and validity.

(a)	CR	AVE	Construct	1	2	3	4	5	6	7	8
0.933	0.957	0.882	1. INC	0.939							
0.931	0.956	0.879	2. INI	0.834	0.938						
0.948	0.960	0.829	3. IQ	0.781	0.808	0.911					
0.946	0.958	0.793	4. LP	0.811	0.812	0.859	0.890				
0.949	0.967	0.908	5. SAT	0.777	0.798	0.817	0.874	0.953			
0.862	0.916	0.784	6. SEQ	0.709	0.770	0.799	0.756	0.710	0.885		
0.905	0.940	0.840	7. SQ	0.773	0.816	0.879	0.837	0.840	0.799	0.917	
0.928	0.955	0.875	8. TTF	0.771	0.774	0.815	0.868	0.846	0.737	0.809	0.936

After confirming the reliability, convergent, and discriminant validity of all study constructs, a quality assessment of the goodness-of-fit for the study model is necessary. In SmartPLS, this can be measured by verifying the acceptable values of the Standardized Root Mean Square Residual (SRMR) and the Normed Fit Index (NFI). As demonstrated in Table 4, the model has a good fit, with SRMR = 0.043 lower than the limit value of 0.08 and NFI = 0.905 above the acceptable level of 0.9, according to Hair et al. (2018) and Henseler et al. (2014). Additionally, the R-square (R2) value's quality assessment measures the model's predictive power by evaluating the endogenous latent variables. The study model's R square is 0.822, indicating an excellent explanatory power of the model, explaining 82.2 % of the factors affecting learning performance using online systems in this research, according to Hair et al. (2011).

**Table 4:** Quality criteria and goodness-of-fit summary.

	Saturated Model	Estimated Model
SRMR	0.043	0.065
NFI	0.905	0.900
	R Square	R Square Adjusted
OLP	0.822	0.821

### 4.2 The structural model

In this phase, the analysis helps to investigate the path coefficient and test the study hypotheses. It shows that information quality (β = 0.395), service quality (β = 0.144), and system quality (β = 0.346) indicate positive significant relationships with the task technology fit, which indicates that those elements are crucial in an e-learning systems and portals lack the quality of knowledge, services, or systems might affect students' usage and hence their learning performance. Moreover, a positive significant influence of task technology fit (β = 0.521), interaction with colleagues (β = 0.151), and interaction with instructors (β = 0.269) have been found to influence students’ satisfaction with using e-learning systems. These findings suggest that e-learning platforms should support students in their learning process online, together with providing them with interaction tools to communicate with their instructors or other students.

In addition, the analysis reveals that both task technology fit (β = 0.453), and students' satisfaction (β = 0.491), have a positive significant effect on learning performance for students who are using online learning systems. Students' satisfaction serves as a positive mediator in the relationship between task technology fit and their learning performance, with a mediation effect of β = 0.255. Thus, both students' satisfaction and task technology fit predict higher usage of e-learning systems, which, in turn, improve student's online learning performance. Hypotheses results, coefficients, t-values, and p-values are demonstrated in (Table 5).

**Table 5:** Hypotheses results.

Hypothesis	Path	Coefficient	t-Value	*p*-Values	Decision
H1	SQ - > TTF	0.346	7.236	0.000	*Supported*
H2	IQ - > TTF	0.395	7.794	0.000	*Supported*
H3	SEQ - > TTF	0.144	3.637	0.000	*Supported*
H4	INC - > SAT	0.151	4.028	0.000	*Supported*
H5	INI - > SAT	0.269	6.733	0.000	*Supported*
H6	TTF - > SAT	0.521	15.528	0.000	*Supported*
H7	TTF - > OLP	0.453	11.436	0.000	*Supported*
H8	SAT - > OLP	0.491	12.686	0.000	*Supported*

**Fig. 2:**
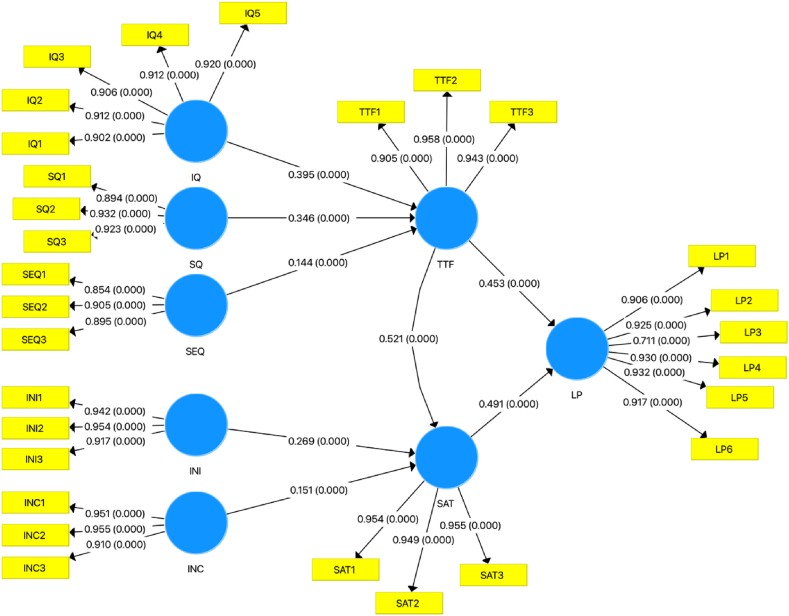
SmartPLS model.

Furthermore, the SmartPLS model for this study is presented in (Fig. 2).

## 5 Discussion

Our study aimed to investigate how the quality of the system, information, and service impacts on TTF. Additionally, it aimed to examine the influence of interaction with colleagues and instructors on students' satisfaction. Furthermore, we explored the effect of TTF on students' satisfaction. Lastly, we investigated how TTF and students' satisfaction affect academic learning performance for students using online learning systems.

The results of our study indicate that system quality has a positive effect on TTF, which contributes to students' perceived compatibility with the technology. Our findings demonstrate that system quality plays a vital role in determining the success of online learning platforms. Our study aligns with previous research that emphasizes the importance of a user-friendly interface to enable easy interaction with the system [26,27]. Furthermore, this leads to a positive user experience. Our study highlights the need to prioritize system quality in online learning platforms to enhance students' learning experiences and performance [32,34]. Learning systems should also be compatible with different devices, such as mobile devices. Additionally, regular maintenance procedures are necessary to ensure error-free versions of the learning system.

Furthermore, the analysis results confirm that the quality of information influences TTF positively. As highlighted in the literature review, one of the important factors for information system success is information quality, as it directly impacts the features of generated information. In fact, high information quality can significantly improve a user's ability to complete assigned tasks, which is consistent with Wu and Chen's (2017) research, and confirmed by further studies conducted [23,32,36,37]. Therefore, information systems need to generate reliable, robust, effective, and relevant knowledge to enhance the user's learning experience and TTF.

Moreover, our analysis confirms that service quality significantly influences TTF, which is aligned with [32,34]. It has been concluded that service quality is crucial in enhancing TTF and improving online learning performance. The support provided by the IT support personnel is essential and necessary to improve the learning performance among system users, which is consistent with [35,38]. Moreover, high service quality can contribute to addressing students’ concerns, maintaining their engagement, and enhancing their learning experience. Prompt technical support, effective communication channels, and aligning assigned tasks with the quality of technologies provided to students are essential in achieving a positive TTF.

On the other hand, our research findings indicate that there is a positive significant influence on the level of interaction among students and their satisfaction with the online learning environment. This result is aligned with previous research works [28,42,46]. Collaborating with other students in online courses enables peer support, diverse perspectives, and collaborative learning. When students engage in discussions, group projects, or social interactions facilitated by the online platform, it creates a sense of community and shared learning experiences. This sense of belonging and connection positively impacts students’ overall satisfaction with their online learning experience. Consequently, higher satisfaction levels improve online learning performance as students are more motivated, engaged, and likely to participate in their courses actively.

Additionally, the interaction between instructors and students plays a vital role in online learning. When instructors provide timely feedback and clear communication and are easily accessible to students, it enhances their satisfaction with the course. Our analysis results indicate that interaction between instructors and students increases their satisfaction with an online learning system, thus improving their online learning performance. Instructors who are highly competent in using technology are best suited to deliver an excellent experience in this interaction. These findings are consistent with [29,55]. Therefore, it is crucial to establish a positive and supportive learning environment through effective interaction between instructors and students.

Regarding the TTF, our analysis confirms the significant positive influence of TTF on Students' Satisfaction. This finding emphasizes the importance of TTF in enhancing user adoption and satisfaction in various industries and technologies [53,54]. In addition, Alyoussef's (2021) study highlights the significance of TTF in influencing student satisfaction and adoption of learning technology [43]. Our finding is also supported by the study of Lin (2012), which found that perceived technology fit, with TTF being a key factor, plays a role in student satisfaction [43,44]. Overall, the significant positive influence of TTF on students' satisfaction emphasizes the extent to which its crucial to align technology with user tasks and needs to ensure a positive user experience and encourage continued use of the system.

Similarly, our analysis confirms the significant positive influence of TTF on online learning performance. This result is consistent with the findings of previous research studies [8,56,57]. These studies have shown that the compatibility between the tasks assigned and the technology used to achieve those tasks is crucial in achieving positive learning performance. The importance of TTF has been emphasized by Alyoussef (2021), who has demonstrated that it strongly influences students’ satisfaction [43]. Furthermore, our findings are consistent with the conclusions drawn by Stefanovic et al. (2016), who have shown that user satisfaction has a positive impact on learning performance within an online learning context [62]. Taken together, our results indicate that TTF is a critical factor in determining online learning performance and should be carefully considered when designing and implementing e-learning systems.

Based on our analysis of student satisfaction and online learning performance, we found that student satisfaction has a significant positive impact on their performance in online learning environments. This finding is in line with previous studies that emphasized user satisfaction's importance in system adoption success and its impact on learning performance (Aldholay et al., 2018; Lin, 2012), which identifies user satisfaction as a critical factor in system adoption success [24,53]. In addition, our findings align with Isaac et al.’s (2017) and Stefanovic et al.’s (2016) conclusions that user satisfaction positively influences learning performance in online learning contexts [61,62]. However, our results are inconsistent with Daud et al. (2011), as they reported no significant impact of user satisfaction on performance [63]. Nevertheless, our results underline student satisfaction's significance in ensuring positive online learning performance, emphasizing TTF's importance when designing and implementing e-learning systems.

Overall, the results of our analysis indicate that several factors significantly influence online learning performance. These factors include system quality, information quality, service quality, interaction among students, interaction between instructors and students, time-to-feedback (TTF), and students' satisfaction. These findings align with previous studies that highlight the importance of a user-friendly interface, high-quality information, effective communication channels, student collaboration, interaction between instructors and students, TTF, and user satisfaction to enhance online learning performance. It is imperative to prioritize system quality to ensure that students have access to reliable, effective, and relevant knowledge. Additionally, prompt technical support must be provided to students to address any issues they may face while using the online learning system. Assigning tasks that align with the quality of technologies available is also essential to ensure that students can complete their work effectively and efficiently. Interaction between instructors and students plays a crucial role in establishing a positive and supportive learning environment. Effective interaction between instructors and students is needed to facilitate communication, provide feedback, and offer academic support. TTF is described by students' use and attitude toward online learning systems and should be taken into consideration when implementing online learning systems to ensure a positive user experience and satisfaction. Encouraging continuous use of the system is vital to ensure that students can continue to benefit from online learning.

## 6 Conclusion

The objective of this study was to investigate the elements that influence university students learning performance utilizing online learning systems. For this purpose, the study investigated several key factors such as the quality of the services and information provided by similar platforms, in addition to the system quality, interaction among students, interaction between instructors and students, TTF, and students' satisfaction with online learning performance. The empirical findings of this study revealed that the quality of the system, information, and service are influencing TTF positively. The results indicate that the system, information, and service of the system are critical determinants of TTF, influencing students' satisfaction and learning performance. Similarly, interaction among students and interaction between instructors and students is revealed to be a substantial predictor of students’ satisfaction.

Together, TTF and students' satisfaction significantly influence online learning performance. Furthermore, they indicated a positive mediation influence on the academic performance of college and university students using online learning systems. To summarize, this study suggests that those designing online learning systems and educators should prioritize the system's quality, information, and service. Therefore, it is essential to prioritize system quality, generate reliable, effective, and relevant knowledge, provide prompt technical support, and align assigned tasks with the quality of technologies. They should also encourage social interaction among students and instructors to improve TTF, satisfaction, and ultimately, academic learning performance, to establish a positive and supportive learning environment through effective interaction between instructors and students to enhance online learning performance.

### 6.1 Research implications

Our investigation carries substantial theoretical and practical ramifications for internet-based learning platforms. Theoretically, this research enhances the existing literature by offering insights into the elements that influence academic performance using online learning systems. The findings of this research underscore importance of user-friendly interfaces, high information quality, effective communication channels, collaboration among students, interaction between instructors and students, TTF, and user satisfaction in enhancing online learning performance. Practically, the study's findings suggest that educators and institutions designing and implementing online learning systems should prioritize user experience, effective support systems, a collaborative learning environment, designing for student needs, and enhancing overall user experience. Additionally, in the post-pandemic era, institutions should focus on flexibility, address the digital divide, and find creative ways to maintain student engagement in online environments.

### 6.2 Limitation and future research work

This study has some limitations that should be considered in future research. First, this study was conducted among undergraduate students, and the findings may not be generalizable to other populations, such as postgraduate students or working professionals. Second, the study was conducted in one university, and the findings may not be applicable to other universities or institutions. Third, the study only focused on the influence of the quality of system, quality of information, quality of service, interaction among students, interaction between instructors and students, TTF, and students' satisfaction on online learning performance, and did not consider other factors such as students’ motivation, prior knowledge, and learning styles. Therefore, future research should focus on investigating the influence of these factors on online learning performance. Additionally, future research could explore the effectiveness of different online learning platforms and technologies in enhancing online learning performance.

## Ethics statement

The research was carried out in compliance with the University of Jordan's protocols and received approval from the Research Ethics Committee (REC) at Luminus Technical University College (Code: LUTC-ETH-500).

## Informed consent

All participants included in the study provided their informed consent prior to participation.

## Data availability

The research data for this study will be accessible from the corresponding authors upon a reasonable request.

## CRediT authorship contribution statement

**Ashraf Bany Mohammed:** Writing – review & editing, Writing – original draft, Supervision, Project administration, Methodology, Investigation, Conceptualization. **Mahmoud Maqableh:** Writing – review & editing, Writing – original draft, Supervision, Project administration, Methodology, Data curation, Conceptualization. **Dhia Qasim:** Writing – original draft, Methodology, Investigation, Formal analysis, Conceptualization. **Faisal AlJawazneh:** Writing – original draft, Methodology, Conceptualization.

## Declaration of competing interest

The authors declare that they have no known competing financial interests or personal relationships that could have appeared to influence the work reported in this paper.

## Figures and Tables

**Table 6:** Operational variable table of the proposed model.

Variable	Definition	Source
System quality	Described as the degree of ease users experience in operating, connecting, and learning systems, along with the level of enjoyment derived from their use.	[85]
Information quality	Described the degree of confidence users place in the precision, comprehensiveness, timeliness, organization, and currency of information obtained from online learning.	[85]
Service quality	Defined by dependability, confidence, physical presence, promptness, engagement, understanding, and effectiveness.	[85]
Task-technology fit	TTF refers to the degree to which systems align with interests, effectively handle tasks, and fulfill requirements.	[25]
User satisfaction	The extent to which users view systems as valuable and want to use them again.	[86]
Interaction with instructors	Refers to opportunities to communicate, exchange and share information, knowledge, materials between students and instructors for the effective construction of students' knowledge through a particular online learning system.	[76]
Interaction with students	Refers to opportunities to communicate and share information, knowledge, and materials between students for the effective construction of their knowledge through a particular online learning system.	[76]
Performance Impact	Described as the degree to which using a system improves the quality of work by facilitating rapid task completion, providing greater job control, enhancing work performance, reducing errors, and increasing work efficiency.	[8]
