# Supplementary material for: Exploring the factors influencing academic learning performance using online learning systems
Source: Heliyon. 2024 Jun 6;10(11):e32584. doi: 10.1016/j.heliyon.2024.e32584 (PMC11193035; doi:10.1016/j.heliyon.2024.e32584)
Supplement: Multimedia component 1 [file mmc1.docx]

| System Quality (SQ) | SYQ1: I find the online learning during COVID-19 Pandemic to be easy to use. |
| --- | --- |
|  | SYQ2: I find the online learning during COVID-19 Pandemic to be flexible to interact with. |
|  | SYQ3: My interaction with the online learning during COVID-19 Pandemic is clear & understandable. |
| Information Quality (IQ) | INQ1: Online learning during COVID-19 Pandemic provides up-to-date knowledge. |
|  | INQ2: Online learning during COVID-19 Pandemic provides accurate knowledge. |
|  | INQ3: Online learning during COVID-19 Pandemic provides relevant knowledge |
|  | INQ4: Online learning during COVID-19 Pandemic provides comprehensive knowledge. |
|  | INQ5: Online learning during COVID-19 Pandemic provides organized knowledge. |
| Service Quality (SYQ) | SYQ1: I could use the online learning during COVID-19 Pandemic services at anytime, anywhere I want. |
|  | SYQ2: Online learning during COVID-19 Pandemic offers multimedia (audio, video, and text) types of course content. |
|  | SYQ3: Online learning during COVID-19 Pandemic enables interactive communication. |
| Task-technology fit (TTF) | TTF1: Online learning during COVID-19 Pandemic fits with the way I like to learn and study. |
|  | TTF2: Online learning during COVID-19 Pandemic is suitable for helping me complete my academic assignments. |
|  | TTF3: Online learning during COVID-19 Pandemic is necessary to my academic tasks. |
| User Satisfaction (SAT) | SAT1: My decision to use the online learning during COVID-19 Pandemic was a wise one. |
|  | SAT2: The online learning during COVID-19 Pandemic has met my expectations. |
|  | SAT3: Overall, I am satisfied with the online learning during COVID-19 Pandemic. |
| Learning performance | LP1: The online learning during COVID-19 Pandemic has a large positive impact on my effectiveness and productivity as a student. |
|  | LP2: Online learning system during COVID-19 Pandemic is an important and valuable aid to me in my studies. |
|  | LP3: I learn better with Online learning system during COVID-19 Pandemic than without it. |
|  | LP4: I can easily achieve the learning goals asserted by the courses with Online learning system during COVID-19 Pandemic. |
|  | LP5: I gain a lot of knowledge with Online learning system during COVID-19. |
|  | LP6: Overall, I learn a lot from the online learning system during COVID-19. |
| Interaction with Instructors | INI1: Using Online learning during COVID-19 Pandemic facilitates interaction with the instructor. |
|  | INI2: Using Online learning during COVID-19 Pandemic is an effective way to communicate with the instructor. |
|  | INI3: Online learning during COVID-19 Pandemic are used effectively to share class materials with the instructor. |
| Interaction with Colleagues | INC1: Using Online learning during COVID-19 Pandemic facilitates interactions with colleagues. |
|  | INC2: Using Online learning during COVID-19 Pandemic is an effective way to communicate with colleagues. |
|  | INC3: Online learning during COVID-19 Pandemic are used effectively to share class materials with colleagues. |
